# BGIR: A Low-Illumination Remote Sensing Image Restoration Algorithm with ZYNQ-Based Implementation

**DOI:** 10.3390/s25144433

**Published:** 2025-07-16

**Authors:** Zhihao Guo, Liangliang Zheng, Wei Xu

**Affiliations:** 1 Changchun Institute of Optics, Fine Mechanics and Physics, Chinese Academy of Sciences, Changchun 130033, China; guozhihao23@mails.ucas.ac.cn (Z.G.); xuwei@ciomp.ac.cn (W.X.); 2 University of Chinese Academy of Sciences, Beijing 100049, China; 3 Key Laboratory of Space-Based Dynamic and Rapid Optical Imaging Technology, Chinese Academy of Sciences, Changchun 130033, China

**Keywords:** remote sensing images, HSV space, image restoration system, ZYNQ, Retinex algorithm

## Abstract

When a CMOS (Complementary Metal–Oxide–Semiconductor) imaging system operates at a high frame rate or a high line rate, the exposure time of the imaging system is limited, and the acquired image data will be dark, with a low signal-to-noise ratio and unsatisfactory sharpness. Therefore, in order to improve the visibility and signal-to-noise ratio of remote sensing images based on CMOS imaging systems, this paper proposes a low-light remote sensing image enhancement method and a corresponding ZYNQ (Zynq-7000 All Programmable SoC) design scheme called the BGIR (Bilateral-Guided Image Restoration) algorithm, which uses an improved multi-scale Retinex algorithm in the HSV (hue–saturation–value) color space. First, the RGB image is used to separate the original image’s H, S, and V components. Then, the V component is processed using the improved algorithm based on bilateral filtering. The image is then adjusted using the gamma correction algorithm to make preliminary adjustments to the brightness and contrast of the whole image, and the S component is processed using segmented linear enhancement to obtain the base layer. The algorithm is also deployed to ZYNQ using ARM + FPGA software synergy, reasonably allocating each algorithm module and accelerating the algorithm by using a lookup table and constructing a pipeline. The experimental results show that the proposed method improves processing speed by nearly 30 times while maintaining the recovery effect, which has the advantages of fast processing speed, miniaturization, embeddability, and portability. Following the end-to-end deployment, the processing speeds for resolutions of 640 × 480 and 1280 × 720 are shown to reach 80 fps and 30 fps, respectively, thereby satisfying the performance requirements of the imaging system.

## 1. Introduction

Remote sensing imagery has important applications in a number of fields, such as military reconnaissance, topographic mapping, meteorological monitoring, environmental protection, and agricultural management. In military applications, remote sensing images are used to track enemy activities and obtain battlefield information; in the field of surveying and mapping, they help generate high-precision maps and terrain models; meteorology uses remote sensing data for weather forecasting and climate change research; environmental monitoring relies on remote sensing to analyze changes in the ecological environment and the impact of natural disasters; and, in the field of agriculture, remote sensing images are used to monitor the growth of crops and the precise application of fertilizers, thus improving crop yield and management efficiency. However, remote sensing images often suffer from low illumination due to insufficient light or exposure, which leads to reduced signal-to-noise ratios, color distortion, and insufficient contrast. Therefore, it becomes necessary to enhance the quality of low-light remote sensing images in order to present image details and scene information more clearly [1].

Traditional image enhancement techniques include methods such as histogram equalization, spatial domain-based convolutional filtering, and gamma correction. Histogram equalization enhances contrast by adjusting the brightness distribution of the image and uses the histogram information of the image to make the gray-level distribution as uniform as possible, thus enhancing details and features. This method is effective when processing low-contrast images but may result in the loss of some details. Spatial filtering processes an image through a convolution operation to reduce noise or enhance edges. Common filters include mean filters (for smoothing noise) and Laplace filters (for enhancing edges). These methods are able to preserve image features while removing unwanted interference. However, although these traditional methods improve image quality to some extent, they usually enhance only one or two aspects of the image, making it difficult to significantly improve the overall visual effect of low-light remote sensing images. Non-physical model enhancement algorithms enhance the visual effect of the image by smoothing low-frequency noise and sharpening high-frequency information to achieve a more desirable low-light image enhancement effect. Representative algorithms include the Retinex algorithm, which was proposed by Land et al. [2] based on the human visual perception model, and the human visual perception model, which simulates the adaptive ability of the human eye to adapt to brightness, color, and contrast, achieving more subjective image processing by separating the illumination and reflection components. It is a classical image enhancement algorithm that is often applied to the enhancement of low-illumination images. The Retinex theory divides the image into two parts, light and reflection, in which the light part corresponds to the low-frequency signal part of the image, and the reflection component corresponds to the high-frequency signal part of the image. Based on Retinex theory, Jobson et al. [3] proposed the single-scale Retinex (SSR) algorithm, which uses Gaussian low-pass filtering to estimate the illumination component, but it suffers from serious color distortion after enhancement and produces an overall whiteness in the image. To address the problem of color distortion, Jobson et al. [4] subsequently proposed the multi-scale Retinex (MSR) algorithm, which uses low-pass filtering with multiple Gaussian kernels to estimate the light component. However, this algorithm does not resolve the color distortion problem and the image is accompanied by the halo phenomenon. Rahman et al. [5] proposed the multi-scale Retinex with color restoration (MS-RCR) algorithm, which uses the color factor C to adjust the ratio of the three RGB channels, thereby reducing color distortion to some extent; however, some distortion remains. Wang et al. [6] proposed using a bilateral filter to estimate the illumination component. The approach improves the halo phenomenon and the computational complexity but does not resolve the color distortion problem. Chen et al. [7] proposed shooting multi-frame images, converting the low-illumination image to the YUV color space, and using a noise reduction method to select the optimal Y-channel component. Huang et al. [8] proposed combining smoothing clustering and the improved Retinex algorithm to estimate the illumination of the low-illumination panorama image enhancement algorithm.

To address the challenge of uneven illumination in images, numerous Retinex-based enhancement methods have been proposed. Extending traditional Retinex theory, Guo et al. [9] proposed an image enhancement method that estimates an illumination map through maximum grayscale value extraction across RGB channels, followed by refinement to produce improved results. For low-light image enhancement, Dong et al. [10] capitalized on dark channel prior-based defogging techniques. Their method exploits the similarity between inverted low-light images and foggy images in grayscale distribution, enabling visibility enhancement through defogging-inspired processing. Hao et al. [11] further advanced this field with a semi-decoupled decomposition (SDD) model, where decomposition is achieved via a pseudo-decoupling scheme: the illumination component is derived from Gaussian variation, while reflectance is jointly computed from the input image and the illumination component. However, existing methods still face limitations. For instance, Cai et al.’s [12] Joint Internal and External Prior (JieP) model, while effective, tends to over-smooth illumination and reflectance components. To mitigate this issue, Li et al. [13] developed a robust structure-aware Retinex enhancement method that incorporates noise mapping, significantly improving the algorithm’s robustness and effectiveness in low-light conditions. Collectively, these contributions refine Retinex theory and expand its applicability in image enhancement.

Currently, there are many other methods to combine deep learning (AI) with low-illumination images. Wang et al. [14] proposed a multi-exposure fusion algorithm for low-light enhancement, leveraging simulated exposure data. Lore et al. [15] demonstrated that a unified deep learning framework could simultaneously achieve low-light enhancement and denoising. Chen et al. [16] designed the Retinex-Net, which includes two parts, Decom-Net and Enhance-Net. Jiang et al. [17] proposed an efficient unsupervised generative adversarial network, including a global–local discriminator structure, self-regularized perceptual loss fusion, and an attention mechanism. Jiang et al. [18] proposed a new degenerate generative network (DRGN), which not only can synthesize enough paired samples for model training, but also effectively solves the complex unmixing problem and improves the visibility of images. However, deep learning methods usually have high computational demands, which makes it difficult to meet the real-time requirements in scenarios with limited hardware resources and certain power consumption constraints (mainly oriented to satellite payload platforms). In recent years, a variety of efficient algorithmic improvement schemes have also been proposed in the field of remote sensing. Xiao et al. [19] proposed SpikeSR, a spiking neural network (SNN)-based approach for achieving efficient remote sensing image super-resolution while maintaining benchmark performance, and Zhang et al. [20] designed a lightweight hybrid neural network to solve the challenges of large model size and high computational complexity in the neural network of remote sensing images. Wang et al. [21] proposed an attention-based multilevel feature fusion network to improve the resolution of RSI, integrating three efficient design strategies to provide a lightweight solution.

In recent years, hardware-accelerated low-light image-processing techniques have made significant progress. Lv et al. [22] innovatively combined bootstrap filtering, grayscale stretching, and image fusion techniques to effectively address low-light defogging challenges. Wang’s team [23] took an alternative approach to develop a lightweight CNN architecture for low-light enhancement, and verified its excellent performance by deploying it on hardware platforms. In the field of target detection, Qu et al. [24] introduced the attention mechanism into the YOLOV4 miniaturized model, achieving performance breakthroughs through FPGA acceleration. For agricultural application scenarios, Luo’s research group [25] proposed a seven-layer Lite CNN which significantly improves the real-time performance of plant disease recognition while maintaining accuracy through model compression techniques such as knowledge distillation. Particularly noteworthy is the low-light defogging system developed by Zhang and other scholars [26] based on the Zynq platform, which employs the HLS toolchain to realize the IP core design of the Retinex algorithm, providing an efficient hardware solution for real-time LCD image processing. Together, these research efforts promote the development of real-time image-processing technology in edge computing environments.

In this paper, a ZYNQ-based hardware solution is proposed to address the problems of blurred edges and insufficient contrast in low-light remote sensing images. The solution adopts the improved BGIR (Bilateral-Guided Image Retinex) algorithm by implementing multi-scale Retinex enhancement in the HSV color space, and this combination not only improves the results of the image enhancement, but also accelerates the performance, which is significantly novel compared with the existing low-light image restoration methods. When converting the image from RGB to HSV space, only the V (value) channel requires processing, and the computational complexity is reduced by 66% of compared to the traditional three-channel processing, which significantly improves the real-time processing capability and is more suitable for real-time application scenarios. Adopting bilateral filtering instead of the traditional Gaussian filtering effectively suppresses the loss of edge information and maintains the integrity of details at the image edge transitions, and gamma correction is introduced at the end of the algorithmic process, which further improves the local gamma correction, to further improve the local contrast, so that the enhanced image is more in line with the visual characteristics of the human eye. Finally, multiple hardware optimization techniques are implemented for the algorithm in the hardware implementation, and a set of complete imaging systems based on OV5640 image sensors is implemented. The results are displayed in real time on the LCD, which provides a reliable platform for the verification of the algorithm.

## 2. Algorithm Overview

### 2.1. BGIR Algorithm Steps

**Figure 1:**
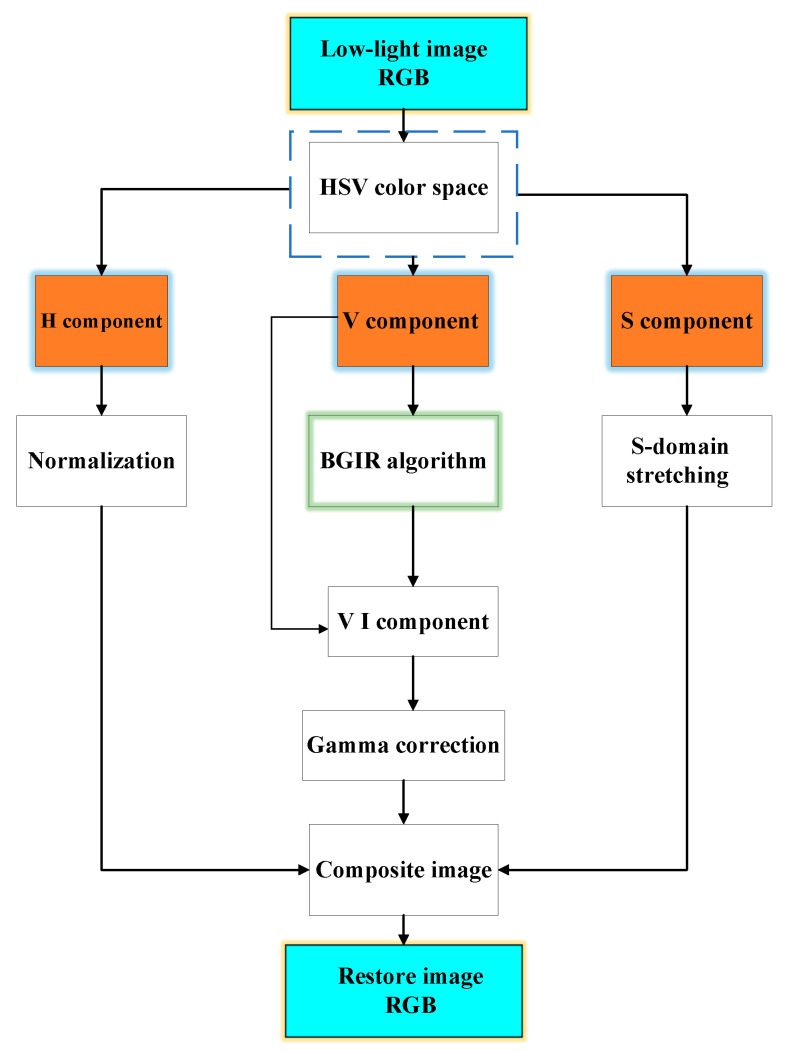
Algorithm flow chart.

To address the shortcomings of the traditional Retinex theory algorithm combined with the HSV space and gamma correction algorithm, and so improve on Retinex algorithm fusion, we put forward a new realization of the algorithm and deploy it to the hardware platform ZYNQ. The algorithm flow is shown in Figure 1, and the specific steps are as follows:

1. The original low-light remote sensing RGB image is converted to HSV space to obtain H, S, and V components.
2. Improved algorithmic enhancement of luminance V is performed to globally improve image clarity and retain image detail information.
3. To improve the local contrast of the image and to improve the visibility of the image to the human eye, the above results are gamma corrected for enhancement.
4. The component H is kept unchanged, and the component S is segmented via linear enhancement.
5. The results are transformed back to the RGB color space, and the restored image is output.

### 2.2. Retinex Theory and Algorithms

**Figure 2:**
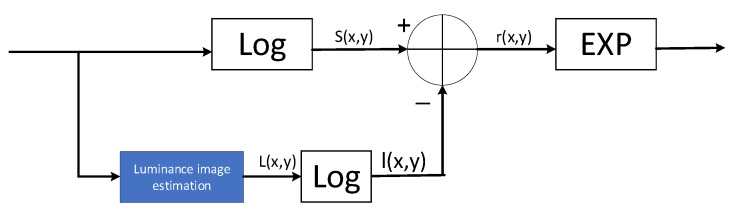
Steps in the single-scale Retinex algorithm.

The Retinex theory is based on the illumination–reflection model of an image, where the reflection component of an image is obtained by removing or reducing the irradiation component of the original image obtained by estimation to obtain a clear image. Its mathematical expression is shown in Equation (1):

(1) $$S\left(x,y\right)=R\left(x,y\right)\times L\left(x,y\right)$$

where x and y denote the coordinates of the pixel points; $S\left(x,y\right)$ denotes the initial image; $L\left(x,y\right)$ denotes the image irradiation component; $R\left(x,y\right)$ denotes the image reflection component. The single-scale Retinex mathematical expressions are shown in Equations (2)–(5):

(2) $$\mathrm{lg}\left[S\left(x,y\right)\right]=\mathrm{lg}\left[L\left(x,y\right)\right]+\mathrm{lg}\left[R\left(x,y\right)\right]$$

(3) $$\mathrm{lg}\left[R\left(x,y\right)\right]=\mathrm{lg}\left[S\left(x,y\right)\right]-\mathrm{lg}\left[L\left(x,y\right)\right]$$

(4) $$L\left(x,y\right)=F\left(x,y\right)\;*\;S\left(x,y\right)$$

(5) $$F\left(x,y\right)=Ke^{\frac{\left(x^{2}+y^{2}\right)}{\sigma^{2}}}$$

where $F\left(x,y\right)$ is the Gaussian convolution function, * denotes the convolution operation, the coefficient K is determined by the normalization function, and σ is the scale parameter of the Gaussian kernel function. The flow of the single-scale Retinex algorithm is shown in Figure 2.

The multi-scale Retinex algorithm can be viewed as the result of a linearly weighted combination of multiple single-scale Retinex algorithms on different scales, with mathematical expressions as shown in Equation (6):

(6) $$R_{i}\left(x,y\right)=\sum_{k=1}^{k}W_{k}\left(\mathrm{lg}\left[S_{i}\left(x,y\right)\right]-\mathrm{lg}\left[F_{k}\left(x,y\right)\;*\;S_{i}\left(x,y\right)\right]\right),\;\;\;i\epsilon \left\{R,G,B\right\}$$

where $R_{i}\left(x,y\right)$ is the output of the single-scale Retinex algorithm; i = 1, …, N, represents the number of spectral bands, where N = 1 represents the grayscale image and N = 3 represents the color image; and k represents the number of Gaussian functions and is generally selected as the three high, medium, and low scales. Usually, k = 3 is taken when k = 1, and the multi-scale Retinex algorithm becomes a single-scale Retinex algorithm. $F_{k}\left(x,y\right)$ represents the scale function, with the Gaussian function usually selected as the scale function. The MSR algorithm will process results at different scales through a weighted combination of color fidelity and balanced image detail information.

MSRCR introduces a color recovery factor (C) to suppress the proportional imbalance between color channels and maintain color naturalness, which is achieved by the formulas in Equations (7) and (8):

(7) $$R_{MSRCR}\left(x,y\right)=C\left(x,y\right)\cdot R_{MSR}\left(x,y\right)$$

(8) $$C\left(x,y\right)=\alpha \cdot \log\left(\frac{\lambda I_{c}\left(x,y\right)}{\Sigma_{c=1}^{3}I_{c}\left(x,y\right)}\right)$$

In the formula, $I_{c}\left(x,y\right)$ represents the pixel value of the cth color channel (R/G/B), and α and λ represent the adjustment parameters.

**Figure 3:**
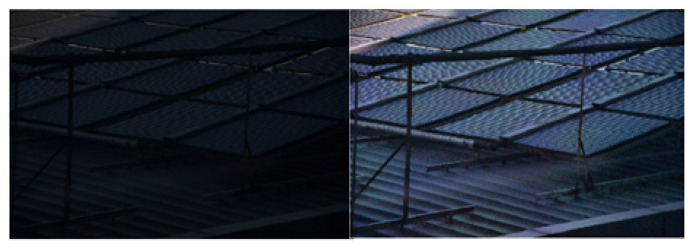
Experiment using the MSR algorithm in the RGB domain.

As illustrated in Figure 3 (image from the LOL [16] dataset), the image that has been processed by the MSR algorithm demonstrates a substantial enhancement in brightness and an improvement in defect visibility. However, the figure also demonstrates that some regions of the image are over-enhanced, resulting in a disruption of the original proportionality of the R, G, and B image components. This phenomenon is accompanied by local exposure issues and a significant loss of detailed information, accompanied by color distortion.

### 2.3. HSV Spatial Domain Transformations

When applying the MSR algorithm in the RGB color space, it is first necessary to process each of the image’s color channels—red (R), green (G), and blue (B)—independently. Specifically, the MSR algorithm enhances the visual effect of the image by adjusting the brightness and contrast of each channel. To achieve this goal, the MSR algorithm uses a multi-scale processing technique, i.e., by blurring the image at various scales to simulate the human eye’s perception of brightness. The image of each color channel is blurred at different scales and undergoes a ratio operation to enhance the brightness and detail performance of the image.

However, even though the brightness and contrast of the image are improved, the MSR algorithm’s processing of each of the three RGB color channels also alters the proportionality between them. While this processing enhances the visual effect of the overall image, it also introduces inconsistencies in the color structure, which is manifested as color distortion. This means that the brightness and contrast adjustments of the brightness and contrast adjustments of the RGB channels are not identical, resulting in color shifts in the image, making the colors appear visually unnatural. Therefore, although the MSR algorithm can improve the quality of low-light images to a certain extent, its impact on the color structure cannot be ignored, and the issue of color distortion becomes particularly evident in the processed images. This phenomenon reminds us that, when applying such algorithms, in addition to brightness and contrast enhancement, the naturalness and coordination of colors need to be taken into account in order to avoid the side effects of over-processing.

The HSV space separates the value from the hue and saturation of the image, allowing Retinex to process the luminance channel (V) directly to avoid color distortion. Only the V channel needs adjustment, and the chromaticity (H, S) remains unchanged to prevent color distortion, which is especially suitable for remote sensing imaging scenarios where the lighting is uneven but the color needs to be preserved. Since HSV only processes the V channel, and the computational load is about one-third of that required for the RGB channels, it is very suitable for real-time applications deployed on the hardware platform.

**Figure 4:**
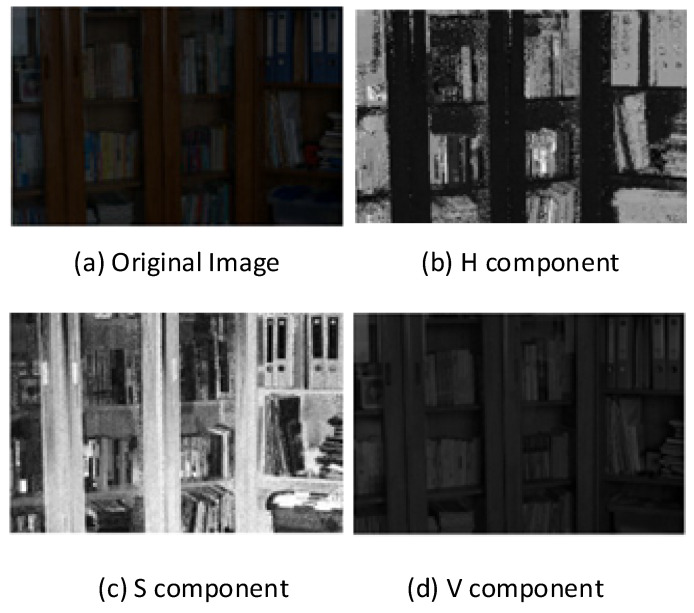
Original diagram and schematic of each component.

In color space analysis, hue represents the type of color through angular measurements ranging from 0° to 360°, where 0° corresponds to red, 120° to green, and 240° to blue, enabling visual color identification. Saturation quantifies color purity on a scale from 0% (completely grayscale) to 100% (fully vibrant), where increased saturation enhances color vividness while decreased saturation shifts towards neutral gray tones, thus allowing for adjustment of color sharpness. Value represents the color brightness or light intensity, similarly scaled from 0% (darkest) to 100% (brightest), with higher luminance values producing brighter appearances and lower values creating darker tones, so that identical hues exhibit different visual characteristics under varying illumination conditions. As illustrated in Figure 4, our proposed algorithm selectively enhances only the luminance component, thereby preserving the original hue and saturation information while improving image visibility. The equations for conversion between RGB and HSV are as shown in (9)–(12):

(9) $$H=\left\{\begin{array}{l} 0\;\;\;\;\;\;\;\;\;\;\;\;\;\;\;\;\;\;\;\;\;\;\;\;\;\;\;\;\;\;\;\;\;\;\;\;\;\;\;\;\;\;\;\;\;\;\;\;\;\;\;\;\;Max=Min\\ \frac{1}{6}\times \frac{G-B}{Max-Min}\;\;\;\;\;\;\;\;\;\;\;\;\;\;\;\;\;\;\;\;\;\;\;\;\;\;Max=R\;and\;G>B\\ \frac{1}{6}\times \frac{G-B}{Max-Min}+1\;\;\;\;\;\;\;\;\;\;\;\;\;\;\;\;\;\;\;Max=R\;and\;G<B\\ \frac{1}{6}\times \frac{B-R}{Max-Min}+\frac{1}{3}\;\;\;\;\;\;\;\;\;\;\;\;\;\;\;\;\;\;Max=G\\ \frac{1}{6}\times \frac{R-G}{Max-Min}+\frac{2}{3}\;\;\;\;\;\;\;\;\;\;\;\;\;\;\;\;\;\;Max=B \end{array}\right.$$

(10) $$S=\left\{\begin{array}{l} 0\;\;\;\;\;\;\;\;\;\;\;\;\;\;\;\;\;\;\;\;\;\;\;\;\;\;\;\;\;\;\;\;\;\;\;\;\;\;\;\;\;\;\;\;\;\;\;\;\;\;\;Max=Min\;or\;V=0\\ \frac{Max-Min}{Max+Min}=\frac{Max-Min}{2V}\;\;\;\;\;\;0<V\leq \frac{1}{2}\\ \frac{Max-Min}{2-\left(Max+Min\right)}=\frac{Max-Min}{2-2V}\;\;V>\frac{1}{2} \end{array}\right.$$

(11) $$V=\frac{1}{2}(Max+Min)$$

(12) $$\begin{array}{c} \left(R,\;G,\;B\right)=\left\{\begin{array}{c} \left(V,\;t,\;p\right)\;\;{\;\;\;\;\;\;h}_{i}=0\\ \left(q,V,\;p\right)\;\;{\;\;\;\;\;\;h}_{i}=1\\ \left(p,\;V,\;t\right)\;\;{\;\;\;\;\;\;h}_{i}=2\\ \left(p,\;q,\;V\right)\;\;\;\;\;\;\;\;h_{i}=3\\ \left(t,\;p,\;V\right)\;\;{\;\;\;\;\;\;h}_{i}=4\\ \left(V,\;p,\;q\right)\;\;\;\;\;\;\;\;h_{i}=5 \end{array}\right.\\ h_{i}=\left[6\times H\right]mod6,\;f=6\times H-h_{i}\\ t=V\times \left(1-\left(1-f\right)\times S\right),\;p=V\times \left(1-S\right),\;q=V\times (1-f\times S) \end{array}$$

where (R, G, B) represents the red, green, and blue coordinates of the pixel point, and the values range from 0 to 1. Max is the maximum value of R, G, and B, and min the minimum value. The corresponding component decomposition diagram is shown in Figure 4 (image from the LOL [16] dataset).

### 2.4. Bilateral Filter Center Surround Function

In this paper, an improved version of the Retinex algorithm is proposed based on the analysis of existing Retinex algorithms. The traditional Retinex framework uses Gaussian linear filtering to estimate the illumination component of an image, thus obtaining an irradiation component that represents the real image. The algorithm is able to achieve good enhancement results provided that the image satisfies the light consistency assumption, i.e., the light intensity in the image remains consistent and varies smoothly. However, this assumption is often not met in practice, particularly in the high-contrast edge regions of the image. When the algorithm estimates illumination, it is limited by the Gaussian filter only based on the pixel distance. This leads to drastic changes in the pixel values of the bright and dark junctions, causing interference, and thus triggering a distortion of illumination estimation and generating halo artifacts, which seriously affects the visual effect. The visual effect is seriously affected. Unlike traditional linear filters, bilateral filtering is a nonlinear filtering method that combines null-domain weights (considering spatial proximity) and value-domain weights (considering grayscale similarity) to smooth an image while effectively preserving edges and details. The bilateral filter kernel degrades to a Gaussian kernel when assuming that there are regions of consistent illumination in the image, while, in the case of inconsistent illumination, the bilateral filter is able to overcome the shortcomings of the Gaussian filter by avoiding excessive smoothing and detail loss. Therefore, the use of bilateral filtering for light estimation can effectively solve the problem of “halo artifacts” in images.

**Figure 5:**
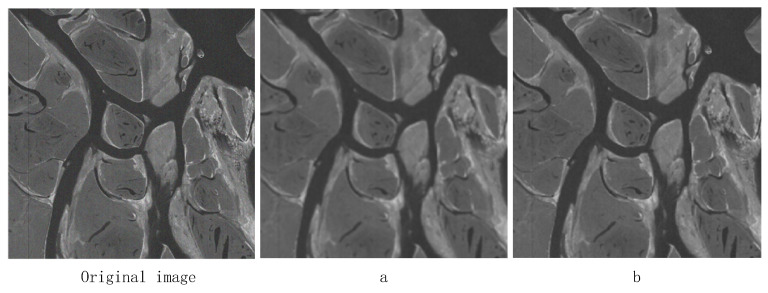
Comparison of Gaussian filtering (**a**) and bilateral filtering (**b**).

A Gaussian filter with the same size window (13), a Gaussian filter with the same value of $\sigma_{s}$ ($\sigma_{s}=4$), and a bilateral filter $\sigma_{r}$ ($\sigma_{r}=0.2$) are used to filter the same image, and the experimental results are shown in Figure 5 (images from our lab), where the a figure shows the Gaussian filtering effect and the b figure shows the bilateral filtering effect.

As shown in Figure 5, the noise in the image is partially suppressed after filtering by both the Gaussian filter and bilateral filter. The edges of the image filtered by the Gaussian filter are blurred and too much image detail information is lost; however, the image filtered by the bilateral filter maintains the edge structure well and the image detail information is more prominent. It is proved that the bilateral filter can maintain the structure of the edges better than the Gaussian filter in low-illumination image enhancement, and the filtering effect is better.

The principle of the bilateral filter used in digital image processing is shown in Equations (13) and (14):

(13) $$f\left(x,y\right)=\frac{1}{w_{p}}\underset{i,j\epsilon \Omega }{\Sigma }w_{s}\left(i,j\right)*w_{r}\left(i,j\right)*R\left(i,j\right)$$

(14) $$w_{p}=\underset{\left(i,j\right)\epsilon \Omega }{\Sigma }w_{s}\left(i,j\right)*w_{r}\left(i,j\right)$$

In the above equations, f(x,y) is the irradiated component of the image with fog, Ω is the neighborhood range of pixel point (i,j), R(i,j) is the original image, $w_{s}\left(i,j\right)$ is the spatial-domain weights, $w_{r}\left(i,j\right)$ is the grayscale-domain weights, and $w_{p}$ is the weighting parameter. The expression is shown by Equations (15) and (16):

(15) $$w_{s}\left(i,j\right)=\exp\left(-\frac{{\left(i-x\right)}^{2}+{\left(j-y\right)}^{2}}{2\sigma_{s}^{2}}\right)$$

(16) $$w_{r}\left(i,j\right)=\exp\left(-\frac{{\left(R\left(i,j\right)-R\left(x,y\right)\right)}^{2}}{2\sigma_{r}^{2}}\right)$$

where $\sigma_{s}$ and $\sigma_{r}$ are the distance standard deviation and gray standard deviation of the Gaussian function, respectively. When the value of $\sigma_{s}$ increases, pixels with large spatial distances can also participate in the filtering, and when the value of $\sigma_{r}$ increases, pixel points with large pixel differences can also participate in the filtering.

Finally, the illuminance component of the bilateral filter is replaced with the original illuminance component of the MSR algorithm to optimize the bilateral filter function and the MSR algorithm.

### 2.5. Gamma Correction

Illumination image estimation is a critical step in the Retinex algorithm. If the illumination component is not accurately estimated, the illumination and reflection images will not be an orthogonal decomposition of the original image, but mixed with each other part of the information, which will lead to the amplification of the error in the subsequent processing, affecting the final processing results. From the perspective of the frequency domain, the illumination image represents the low-frequency part of the original image, which reflects the total intensity of the light source and determines the dynamic range of the image. Due to uneven illumination, the actual image may exhibit low contrast or saturation distortion in certain regions, at which time the bilaterally filtered illuminance image may show lower brightness in some regions. Therefore, the illumination component must be compensated. In this paper, the compensation is performed using gamma correction, a method that aligns with human visual perception, so that the corrected illuminance image is more in line with the assumptions of the Retinex theory. The formula for gamma correction is shown in Equation (17):

(17) $$T\left(I\right)=I_{max}{\left(\frac{I}{I_{max}}\right)}^{\gamma }$$

where I and $I_{\max}$ are the gray value and the maximum gray level of the input image pixels, $T\left(I\right)$ is the changed pixel value, γ is the control parameter in the transformation process to determine the enhancement effect, and γ is the correction factor. When γ > 1, the overall gray value of the image becomes smaller, i.e., the image becomes darker; when γ < 1, the overall gray value of the image becomes larger, i.e., the image becomes brighter. In order to avoid image darkening caused by underestimation of transmittance, the value of γ is set to [0, 1]. According to the data test, γ = 1/2.2 is the better result.

### 2.6. Handling of Saturation Components and Color Recovery

During image enhancement, the saturation (S) of an image usually decreases as the brightness (V) increases. In order to avoid distortion of the image colors and to maintain the sharpness of the colors, saturation needs to be enhanced appropriately. Saturation reflects the purity of the colors, and it must be enhanced in such a way that the enhancement is natural and does not produce noticeable distortion. Excessive grayscale changes can make the image color look unrealistic, so you need to avoid the use of too intense processing methods. In order to obtain a softer and more vibrant color effect, we, according to the original image’s saturation of different levels of saturation, divide saturation into three intervals: the saturation of the higher part of the moderate maintenance or attenuation and the saturation of the lower part of the enhancement. This can effectively enhance the color expression of the image. To realize this saturation enhancement, we use the segmented logarithmic transformation method, as shown in Equation (18):

(18) $$S_{1}\left(x,y\right)=\left\{\begin{array}{l} k_{1}\cdot S\left(x,y\right)\;\;\;\;\;\;\;\;\;\;\;\;\;\;\;\;\;\;\;\;\;\;0\leq S\left(x,y\right)<S_{a}\\ k_{2}\cdot \left[S\left(x,y\right)-S_{a}\right]+a_{1}\;S_{a}\leq S\left(x,y\right)<S_{b}\\ k_{3}\cdot \left[S\left(x,y\right)-S_{b}\right]+a_{2}\;S_{b}\leq S\left(x,y\right)\leq 1 \end{array}\right.$$

where ${\;k}_{1}$, $k_{2}$, and $k_{3}$ are stretching coefficients, $S_{a}$ = 0.3, $S_{b}$ = 0.6, $a_{1}=k_{1}\cdot S_{a}$, and $a_{2}=k_{2}\cdot \left(S_{b}-S_{a}\right)+a_{1}$. When the image saturation is too low, the stretching coefficient selects a larger value; conversely, a smaller value is selected.

## 3. Hardware Design of the BGIR Algorithm

### 3.1. Imaging System Platform Construction

**Figure 6:**
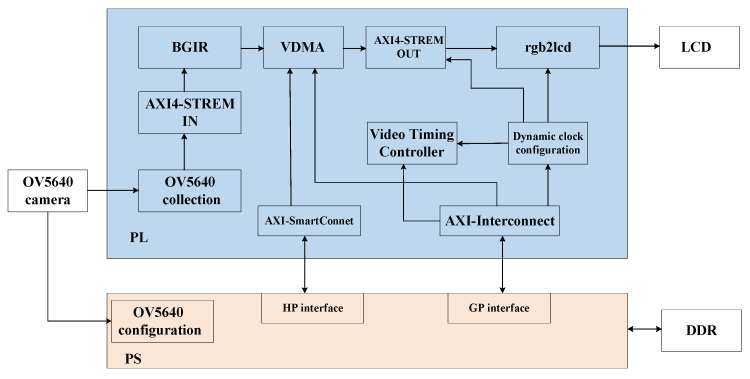
Imaging system construction.

This paper uses the ZYNQ 7020 development board from Xilinx, Inc. (San Jose, CA, USA) as the hardware platform. The OV5640 camera (OmniVision Technologies Inc., Santa Clara, CA, USA) is used to capture images, the off-chip DDR memory caches the images, and the LCD interface monitor (Zhengdian Atom, Guangzhou, China) displays the images. The hardware development and simulation software is Vivado 2018. Figure 6 shows the overall architecture of the ZYNQ image-processing system used in this paper, which is mainly divided into three parts: image acquisition, image processing (integrated into the optimization algorithm IP), and image display.

In the PL-side processing logic, the image-processing pipeline begins with AXI-Stream formatted data being received through the input interface. The data undergoes sequential transformations, starting with conversion from AXI-Stream to hls::Mat format using the hls::AXIvideo2Mat function. A color space conversion follows, where the hls::CvtColor function transforms the image from RGB to HSV representation. The hls::Split function then decomposes the HSV image into its three constituent channels: hue (H), saturation (S), and value (V). The value channel receives specialized processing through the fast_bilateral_filter function, with core luminance enhancement performed by the loop_process function. After applying gamma correction and other adjustments, the modified channels are recombined into an HSV image. The pipeline concludes with inverse color space conversion back to RGB using hls::CvtColor and final formatting to AXI-Stream via hls::Mat2AXIvideo. The whole process is performed in the PL section. The PS-side processing logic, which is chiefly responsible for system-level control and configuration tasks, includes initialization of the EMIO, AXI GPIO, and camera OV5640, configuration of the AXI VDMA, and initialization and configuration of the display controller.

**Figure 7:**
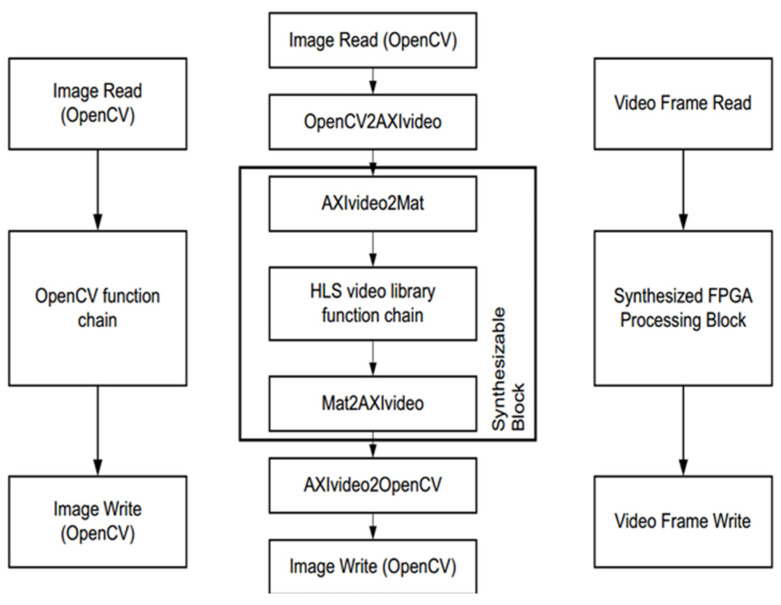
HLS OpenCV development process.

The function of HLS is simply to convert a C or C++ design into an RTL implementation that can then be synthesized and implemented in the programmable logic of a Xilinx Zynq chip. It is important to note that we are talking about a design achieved in C/C++ as opposed to software code running in a processor (ARM processor in ZYNQ or MicroBlaze soft core processor). In HLS, all C designs are implemented in programmable logic, i.e., we are still designing in hardware, but no longer using a hardware description language. The Vivado HLS includes a set of C libraries (in both C and C++) that facilitate the modeling of common hardware structures or functions in C/C++ and synthesize them into RTL. The HLS video library is not exactly the same as the OpenCV library, although many of the video concepts and function names are similar. Since OpenCV functions cannot be synthesized directly, they must be replaced by synthesizable library functions. The main reason for this is that OpenCV functions involve dynamic memory allocation, such as when constructing a cv::Mat object of arbitrary size in OpenCV, and such functions are not synthesizable. The differences are shown in Figure 7.

### 3.2. The Design and Optimization of the IP Core for BGIR

#### 3.2.1. IP Core Design

**Figure 8:**
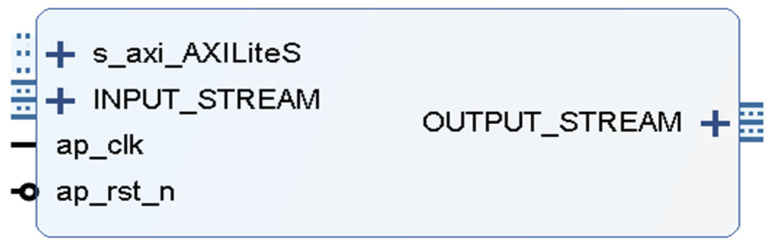
BGIR IP core design.

The BGIR algorithm has been developed based on a pipeline structure, which subdivides each step into multiple stages. This approach facilitates continuous data processing through the utilization of pipelining, which enables the execution of each stage in parallel with the preceding one. This, in turn, results in an enhancement in overall processing efficiency and a reduction in computational latency. Furthermore, BGIR speeds up processing by optimizing data input and output procedures, reducing memory access and data transfer delays, and more. By reducing the overamplification of background noise and accurately preserving picture features during the enhancement process, the BGIR algorithm improves the image’s visual quality. BGIR benefits from fine-grained hardware resource optimization when it is deployed on an FPGA platform, particularly in crucial processing stages (color separation and picture brightness). To increase the accuracy of the results, a high-precision mathematical calculation module is employed. The IP core may be created and utilized in Vivado following the successful synthesis and simulation of the algorithm in HLS. The IP core generated by this algorithm is shown in Figure 8, and the input and output use the AXI4-STREAM interface, the ap_rst_n and ap_clk are used as reset and clock inputs, respectively, and the INPUT_STREAM is used for the ov5640_capture_data module to obtain the images captured from the OV5640 camera through the Video In to AXI4-OUTPUT_STREAM, which is the video stream format of the OV5640_capture_data module. INPUT_STREAM is the video stream format of the ov5640_capture_data module, which acquires the image captured by the OV5640 camera through the Video In to AXI4 Stream module, and OUTPUT_STREAM is the video stream format. This includes information such as the number of rows and columns.

#### 3.2.2. Convolution and Lookup Table Optimization Design

The goal of this algorithm is to be implemented on a hardware platform. However, the MATLABR2018 calculations above involve floating-point operations, which are not ideal for FPGA implementation. Additionally, the Gaussian function has small numerical values, and direct quantization would significantly increase resource usage. Since convolution is a linear operation, the Gaussian kernel can first be scaled up according to Equation (19):

(19) $$G_{\sigma_{r}}\left(\left\|I_{p}-I_{q}\right\|\right)=e^{-\frac{{\left(I_{p}-I_{q}\right)}^{2}}{2\sigma_{r}^{2}}}=e^{-\frac{{\left|I_{p}-I_{q}\right|}^{2}}{2\sigma_{r}^{2}}}$$

**Figure 9:**
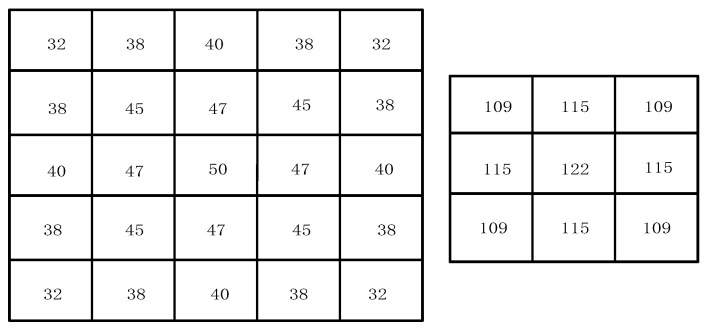
Gaussian function fixed-pointing matrix.

It is easy to see that the absolute value of the difference between two neighboring pixels must belong to [0, 255]; therefore, we can calculate in advance and use fixed-point so that we can use the lookup table method to achieve the pixel similarity weight calculation, as well as use this method later more than the use of floating-point to fixed-point. Finally, the value of the Gaussian function quantized into fixed-point is stored as an initialization file in the form of rows, which is directly solidified in the form of ROM into the Block Random Memory (Block RAM, BRAM) of the FPGA, and the quantization matrix is shown in Figure 9.

**Figure 10:**
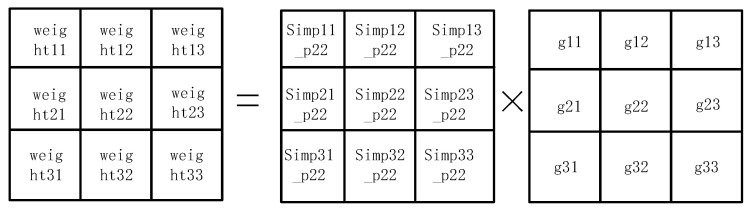
Bilateral filter weight calculation process.

During image processing, we need to calculate the similarity weights within a 3 × 3 window centered on the current pixel. First, for each pixel within this window, we calculate the absolute difference between the pixel value and the center pixel. This difference represents the difference in color or brightness between the two, usually measured using the gray value or color component of the pixel. Once the difference between each pixel and the center pixel is computed, we refer to a predefined lookup table (LUT) to obtain the corresponding similarity weight. The similarity weights of the pixels in the 3 × 3 window are derived separately, with each weight reflecting the degree of similarity between the pixel and the center pixel. A higher weight value indicates that the pixel is more similar to the center pixel. In this way, we are able to assign a corresponding weight to each pixel, so that, in subsequent filtering operations, we can more accurately retain regions of the image with similar features while reducing the influence of regions that are less similar to the center pixel. These calculations are an important part of the bilateral filtering algorithm and can help remove image noise while preserving image details and edge information. The Gaussian weights and similarity weights within the 3 × 3 window are computed. These two weights reflect the spatial distance between pixels and the similarity between pixels, respectively. To combine these aspects, we multiply the Gaussian weight corresponding to each pixel in the 3 × 3 window by its corresponding similarity weight on an element-by-element basis. In this way, we obtain a new weight matrix, as shown in Figure 10, which takes into account not only the geometric distance between pixels (represented by the Gaussian weights), but also the color or brightness similarity between pixels (represented by the similarity weights). The result of this weighting will help to realize bilateral filtering, which can effectively remove noise while maintaining the edge information of the image.

**Figure 11:**
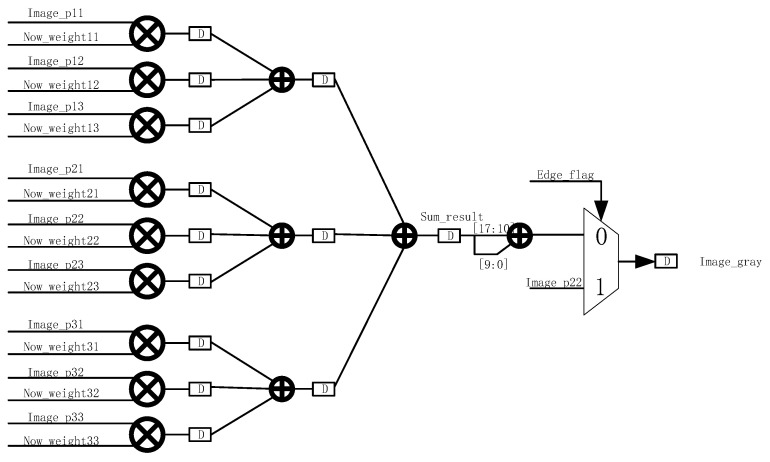
Weights are convolved and accumulated.

During the computation process, an accumulative result is first obtained, denoted as sum_result, which represents the sum of the convolution operation. In order to accurately represent the summed value, sum_result is divided into integer and decimal parts, where sum_result [17:10] represents the integer part and sum_result [9:0] is the decimal part. In order to obtain further filtering results, we need to round sum_result. The purpose of rounding is to convert the sum result into an integer value to ensure that the filtered image is smooth and free from excessive errors. This operation forms the final filtered result sum_result [17:10] + sum_result [9:0] by combining the integer and decimal parts, as shown in Figure 11.

However, in actual image processing, special attention must be given to the boundary situation of the window. If the center pixel of a 3 × 3 window is at the edge or corner of an image, then its surrounding pixels cannot completely form a 3 × 3 window. In this case, in order to avoid boundary-crossing problems in the calculation, we directly output the value of the current center pixel as the filtering result without complex convolution calculation. This method effectively avoids the problems caused by boundary effects and ensures smooth image processing in the edge regions, and will not lead to the distortion of or error in the calculation result due to the edge problem.

**Figure 12:**
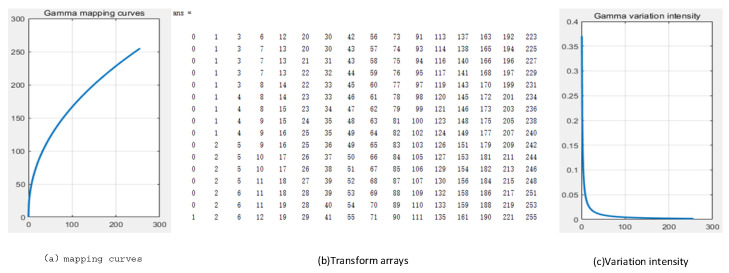
Gamma correction matrix and pixel transformation intensity.

When dealing with large numbers of data, the computation is very slow, making it difficult to satisfy real-time demands and resulting in a significant waste of computational resources. This is due to the fact that direct computation of gamma correction entails applying the gamma function to every image element, which is potentially quite straightforward. Lookup table techniques pre-calculate and store all potential input and output values, which helps expedite the gamma correction process. Since the range of pixel values in 8-bit image processing is 0 to 255, this paper creates a lookup table with 256 elements. This method reduces computation while increasing processing speed because it only requires basic indexing and no sophisticated mathematical operations when applying gamma correction to an image. The gamma correction matrix as well as the pixel transformation intensity is shown in Figure 12.

#### 3.2.3. HLS Command Optimization

**Figure 13:**
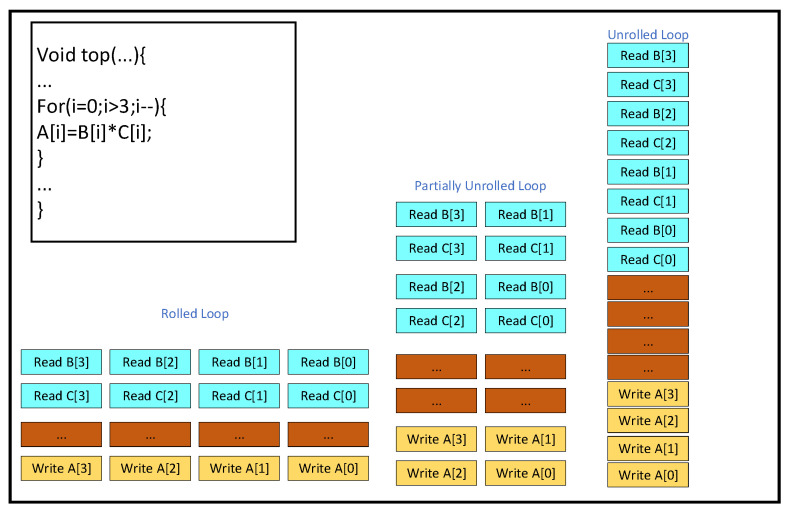
Loop unrolling optimization.

UNROLL is a High-Level Synthesis (HLS) optimization directive that expands a loop, creating multiple independent operations. This results in the parallel execution of operations within the loop during a single clock cycle, in contrast to processor-based architectures, where operations are executed serially. The fact that data can be processed in parallel within the FPGA demonstrates the advantages of parallel acceleration with FPGAs. The “Rolled Loop” on the left is a rolling loop, which means that each iteration is executed in a separate clock cycle, which takes four clock cycles to implement and can be accomplished with a single multiplier in the FPGA. The “Partially Unrolled Loop” in the middle is a rolling loop, which means that each iteration is executed in a separate clock cycle. The “Partially Unrolled Loop” in the middle is a partially expanded loop. In this example, the implementation takes two clock cycles and requires two multipliers in the FPGA. The “Unrolled Loop” on the right is an expanded loop. In this example, the loop is fully expanded and we can use the multiplier in a single clock cycle. In this case, the loop is fully expanded, and we can perform all loop operations in a single clock cycle. However, this implementation requires four multipliers, and, more importantly, it needs to be able to perform four reads and four writes in the same clock cycle, and, since there are only two ports in the FPGA’s Block RAM at most, we need to partition the array when implementing this. It is important to note that, after unrolling the loop, if operations within an iteration depend on the result of the previous iteration, they cannot be executed in parallel. Instead, these dependent operations must be executed sequentially, as soon as the necessary data becomes available. This is shown in Figure 13 below.

**Figure 14:**
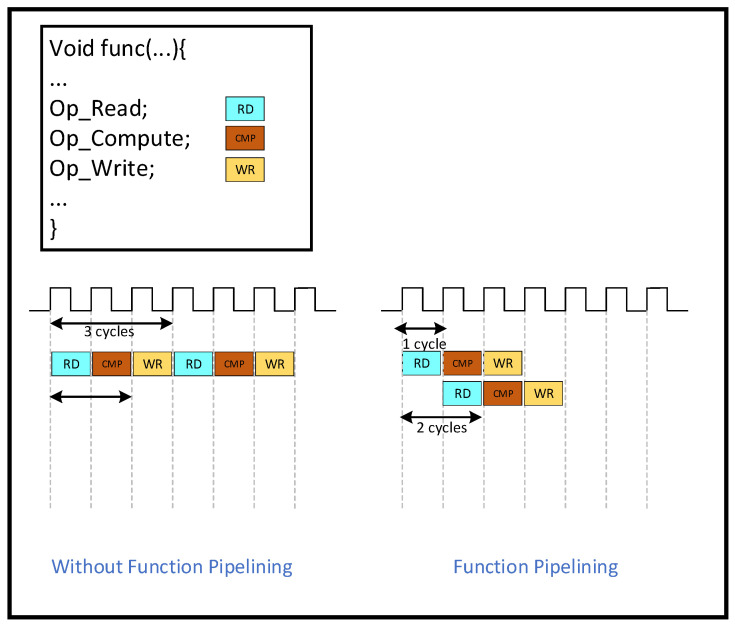
Function pipelining.

PIPELINE is an optimization to increase throughput. “PIPELINE” refers to pipelined operations, which allow operations to occur simultaneously so that a task does not have to complete all operations before starting the next one. Pipelining can be applied to functions and loops. Without pipelining, this function reads an input every three clock cycles and outputs a value every two clock cycles. This function has an initiation interval of 3 and a latency of 2. With pipelining, a new input is read every cycle (initiation interval = 1) without changing the output latency or the resources used. The throughput improvement of the function is shown in Figure 14 below.

**Figure 15:**
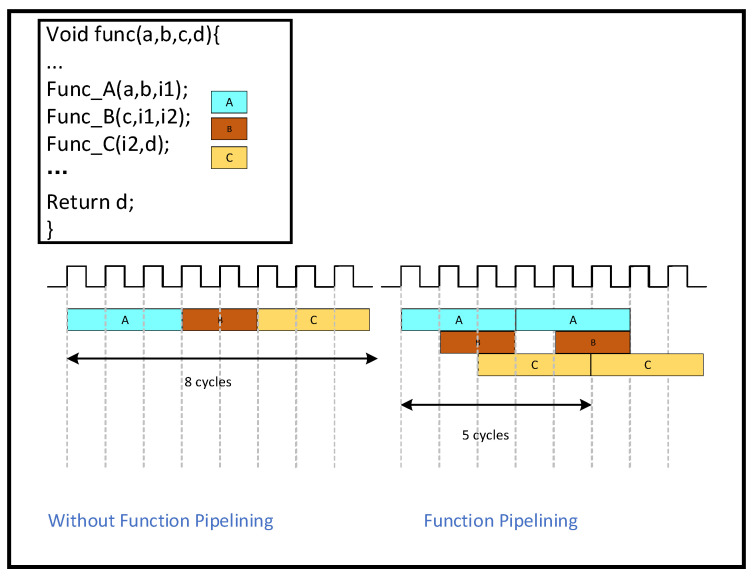
Implementation of dataflow operations.

The DATAFLOW instruction indicates that functions and loops can overlap during execution to form a pipelined structure. This technique can achieve highly parallel operation and greatly increase the algorithm’s execution efficiency. Simultaneously, it can boost the algorithm’s execution efficiency, decrease overall latency, and lessen dependencies between functions and loops. The principle of DATAFLOW execution is shown in Figure 8. Before applying DATAFLOW, A, B, and C are executed serially; we need to wait for A to finish before we can start executing B, and we need to wait for B to finish before we can execute C. As soon as DATAFLOW is applied, B will begin running as long as A is producing something. This enables various activities to overlap, effectively reducing latency and increasing throughput, as shown in Figure 15 below.

### 3.3. System Configuration

**Figure 16:**
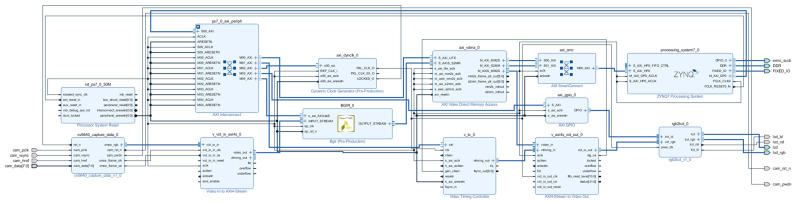
Overall system structure.

This design’s Vivado block body is seen in Figure 16. There is a detailed description of the IP core’s configuration information.

VDMA IP core: The number of frame buffers is set to three to prevent read and write channels from occurring simultaneously, and the address is set to 32 bits to allow for read and write channels for VDMA. The RGB888 read-in data is represented by the stream data width of 24, which is set to 24. Selecting Independent Clocking and setting it to Slave Mode lowers the backpressure on the upstream host and lowers downstream latency. AXI-STREAM to Video Output IP core: The buffer depth is set to 1024 to guarantee that the input and output data rates are approximately equal. The AXI4 Lite interface is added to the VTC IP core, allowing the PS side to configure the VTC to regulate the production of various timed messages. The rgb2lcd IP core implements the capabilities of reading LCD ID and encapsulating multiple LCD interfaces. We use the RGB888 format to set the red, green, and blue depths to 8-, 8-, and 8-bit widths, respectively. The AXI4 stream’s 24-bit data output to the visual output matches the input data bit width of 24 bits. To send the RGB2LCD ID to the PS side, the axi_gpio IP core is set up as an input type.

## 4. Algorithm Experiments as Well as Hardware Experimental Analysis

### 4.1. Experimental Platforms

In order to confirm that the algorithm is successful, the evaluation indexes used in this paper are the Peak Signal-to-Noise Ratio (PSNR) and information entropy. The experimental software environment is a Windows 11 system, and the experimental hardware is configured with an AMD R9 7945HX 2.40 GHz CPU, 12 GB memory, and ZYNQ7020 of the Xilinx company (San Jose, CA, USA). The experiments are mainly conducted through Matlab R2018 to jointly verify the algorithms in software and hardware, and finally via real-time verification on the ZYNQ board.

### 4.2. Evaluation Indicators

Peak Signal-to-Noise Ratio (PSNR) is one of the most common and widely used objective evaluation indexes for images. The larger the PSNR value, the less intrusive and distorted the noise is to the image, which can be expressed as shown in Equation (20):

(20) $$R_{PSNR=10\cdot \mathrm{lg}}\frac{\sum_{i=1}^{M}\sum_{j=1}^{N}{\left\{\max\left[f\left(i,j\right)\right]\right\}}^{2}}{\sum_{i=1}^{M}\sum_{j=1}^{N}{\left\{f^{\prime }\left(i,j\right)-f\left(i,j\right)\right\}}^{2}}$$

where M denotes the height of the image; N denotes the width of the image; $\max\left[f\left(i,j\right)\right]$ denotes the maximum pixel value of the image; $f\left(i,j\right)$ denotes the pixel value of the original image in the i-th row and j-th column; and $f^{\prime }\left(x,y\right)$ denotes the pixel value of the processed image in the i-th row and j-th column.

Information entropy is a measure of the amount of information and uncertainty in an image. The information entropy of an image can be roughly described as the degree of confusion of the image, which is also a concentrated reflection of the amount of information in the image. The larger the entropy, the more complex the information in the image and the richer the details, while, the smaller the entropy, the simpler the single information in the image. The concept of information entropy originates from information theory, proposed by Claude Shannon. Entropy represents the average uncertainty of information in a system. In image processing, information entropy is often used to evaluate the complexity and level of detail of an image. It can be expressed as shown in Equation (21):

(21) $$E=-\sum_{i=0}^{255}P_{i}\left(\log P_{i}\right)$$

By contrasting the brightness, contrast, and structure of the reference and assessed images, the SSIM assesses the quality of the images. Equation (22), which shows the mean values of x and y, where $\delta_{xy}$ is the covariance of x and y, $\delta_{x}^{2}$ and $\delta_{y}^{2}$ indicate the variances of x and y, and $c_{1}$ and $c_{2}$ are constants to provide stability, computes the similarity:

(22) $$SSIM\left(x,y\right)=\frac{\left(2u_{x}u_{y}+c_{1}\right)\left(2\delta_{xy}+c_{2}\right)}{\left(u_{x}^{2}+u_{y}^{2}+c_{1}\right)\left(\delta_{x}^{2}+\delta_{y}^{2}+c_{2}\right)}$$

The contrast of an image is a measure that describes the difference in brightness between adjacent areas in an image, and is the degree to which the gray levels in an image vary. The higher the contrast ratio, the more obvious the difference in brightness in the image, and the image looks sharper and deeper, while a lower contrast ratio means that the difference in brightness in the image is smaller, and the image may appear flatter or blurrier. The formula for calculating the contrast ratio is as shown in Equation (23):

(23) $$CI=\frac{I_{\max}-I_{\min}}{I_{\max}+I_{\min}}$$

CI represents the contrast, Imax represents the maximum luminance of the neighboring image, and $I_{\min}$ represents the minimum luminance of the neighboring image. The local contrast of a color RGB image is calculated as shown in Equation (24) below:

(24) $$CI=\frac{1}{3}\left(CI_{R}+CI_{G}+CI_{B}\right)$$

where $CI_{R}$, $CI_{G}$, and $CI_{B}$ are the local contrast of the R, G, and B components of the image, respectively, and the improved local contrast of each algorithm is used as an objective evaluation parameter to measure the quality of the image compared to the original value (IPCI).

The NIQE (Natural Image Quality Evaluator) is a reference-free image quality evaluation metric for assessing the degree of distortion in natural images without relying on the original image as a reference. It is based on the statistical properties of images and is especially suitable for real scenes where the original image is not available.

The EPI (Edge Preservation Index) is a metric used to evaluate the edge preservation ability of image-processing algorithms (e.g., denoising, super-resolution, compression, etc.). It measures how well an algorithm preserves the key structures (e.g., edges, texture) of an image by comparing the similarity of the edge information between the processed image and the original reference image. The EPI is particularly important in the fields of medical imaging, remote sensing image enhancement, and so on.

(25) $$EPI=\frac{\Sigma \left(G_{ref}-\mu_{ref}\right)\left(G_{proc}-\mu_{proc}\right)}{\sqrt{\Sigma {\left(G_{ref}-\mu_{ref}\right)}^{2}\Sigma {\left(G_{proc}-\mu_{proc}\right)}^{2}}}$$

In the equation, $G_{ref}$ and $G_{proc}$ are the gradient magnitude plots of the reference image and the processed image, respectively, and $\mu_{ref}$ and $\mu_{proc}$ are the mean values of the gradient plots, respectively.

### 4.3. Algorithm PC Comparison Experiment

**Figure 17:**
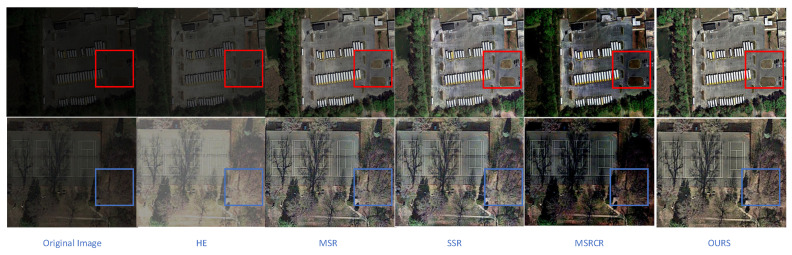
Image enhancement results of various algorithms (comparison targets are in the box).

To verify the effectiveness of the BGIR algorithm, this paper conducts a comparative experiment on a software platform using the HE, MSR, SSR, and MSRCR algorithms for image processing. Figure 17 (image from our lab) presents the image enhancement results of different algorithms, while Table 1 compares the evaluation metrics for the processed images. In Figure 17, the results are labeled sequentially from (a) to (f): (a) represents the original image, (b) corresponds to the HE algorithm, (c) shows the SSR algorithm output, (d) displays the MSR algorithm result, (e) denotes the MSRCR algorithm output, and (f) illustrates the image enhanced using the proposed algorithm in this paper. From the figure, it can be clearly observed that, although the HE (histogram equalization) algorithm enhances the overall brightness of the image to a certain extent, it causes significant loss of color information in the detail region, which negatively impacts the realism of the image. The MSR (multi-scale Retinex) algorithm and SSR (single-scale Retinex) algorithm, on the other hand, overexpose some regions during processing, while the detail information in the edge mutation region becomes blurred, reducing the clarity of the image. The MSRCR algorithm, when applied to remote sensing low-illumination images, is even triggering. The MSRCR algorithm, when applied to remote sensing low-light images, even causes serious color distortion problems, resulting in image colors deviating from the real scene. In contrast, the BGIR algorithm not only significantly enhances the brightness of the image, which is more in line with the visual perception of the human eye, but also retains the detailed information of the image, and the overall enhancement effect is more natural and balanced.

We selected 20 low-light remote sensing images segmented as 1280 × 720 from the DOTA dataset [27] for the experiment, and the average values of specific parameters are shown in Table 2.

Table 1 and Table 2 in this work compare the processing outcomes of the BGIR algorithm for photos of varying resolutions (1280 × 720 and 640 × 480). The results show that the BGIR algorithm with bilateral filtering outperforms the traditional Retinex algorithms (SSR, MSR, MSRCR) and histogram equalization (HE) in terms of the image enhancement task and PSNR, as well as the indexes such as the SSIM.

The specific performance is as follows: PSNR: BGIR reaches 25.4, significantly higher than MSRCR (19.8) and HE (16.2), indicating that its reconstructed image has the smallest error relative to the real image. SSIM: BGIR’s 0.88 is also better than the other algorithms (0.75 for MSRCR and only 0.52 for SSR), demonstrating that it better preserves the structural information of the image. The BGIR algorithm outperforms the HE, SSR, and MSR algorithms in terms of NIQE metrics and is generally comparable to the MSRCR algorithm. In terms of EPI metrics, this paper’s algorithm outperforms the other algorithms across the board, indicating that it has better edge preservation. Applying this algorithm with ZYNQ using fixed-point data may result in lower corresponding information entropy. In addition, gamma correction can compress the grayscale range and further reduce the information entropy. Comprehensively analyzing the data in Table 1 and Table 2, it can be seen that it leads to a decrease in the corresponding information entropy; however, for the resolution adaptation from 640 × 480 to 1280 × 720, the PSNR of BGIR fluctuates (<0.5), and the SSIM remains stable (0.88→0.89), indicating that it is robust to changes in resolution. The other algorithms (e.g., MSR) have a significant decrease in the PSNR under high resolution (15.0→14.6), which exposes the noise sensitivity. The experimental results demonstrate that BGIR is ideal for high- and low-resolution image enhancement, especially for medical imaging, remote sensing, and other scenarios with stringent requirements for detail preservation.

This study carries out experiments comparing the processing speed of the BGIR algorithm with similar algorithms like the MSR and SSR on a PC platform in order to successfully confirm the enhanced processing speed of the BGIR method. The experiment’s resultant data is displayed in Table 3 with clarity. When compared to other algorithms, the BGIR algorithm in fact achieves a pretty significant improvement in processing speed, as demonstrated by an intuitive study of the data in the table.

**Figure 18:**
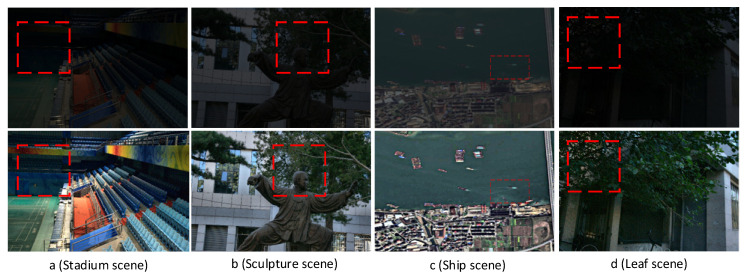
Algorithmic processing results for different scene images (comparison targets are in the box).

This study investigates several types of low-brightness photos on a PC platform to demonstrate how the BGIR algorithm can be generalized. Figure 18 (original image from the LOL [16] dataset) displays the results of the experiment. The four different types of original photos that we used are shown in the a, b, c, and d figures. The image processed by the method in this paper is shown in the following figure. It is made evident by comparing the photographs before and after processing that the processed image has significantly improved brightness and that the fine details have been preserved. In Figure 18a, the color transition between the paint and the wall in the seating area is natural, and the overall hue remains highly consistent without noticeable distortion or blurring. Figure 18b vividly shows the fine texture of the character sculpture; both the facial contour and the clothing carving creases are clearly discernible, while the branches of the trees in the background are clearly layered and rich in details. In the remote sensing image of Figure 18c, the previously blurred outline of the small ship hull is significantly enhanced. The contrast between the sea surface and the ship is improved, and target recognition is substantially better. In Figure 18d, the algorithm achieves accurate retention of the veins of the tree leaves and the subtle features of the surrounding environment, and the overall image maintains the sense of natural realism while highlighting the key information. This series of experimental results fully proves that the BGIR algorithm demonstrates excellent detail retention and enhancement capabilities when dealing with diverse scenes, and has wide applicability and robustness.

We validate this using the full LOL [16] dataset and compare it with previously published experimental results to confirm the overall effectiveness of the BGIR technique developed in this paper. The LOL dataset’s images are 400 by 600 pixels in size, and Table 4 displays the comparison findings. Reference [28] used Gaussian filtering under HSV color, which optimized the algorithm computation but did not completely solve the problem of the blurring of details caused by the algorithm. In reference [29], a bootstrap filter is used instead of a Gaussian filter to optimize the color distortion and detail blurring problem, but it is not ideal for noise. The algorithm in reference [30] is computationally more complex and not easily portable to hardware platforms. References [9,31] improved the denoising variant framework to achieve low-illumination image enhancement. As can be seen from the table, this paper’s algorithm is comprehensively ahead of the algorithm in reference [28]. Compared with other algorithms, it is slightly insufficient in terms of the information entropy, but, in terms of the PSNR index and SSIM index, it is excellent, and the algorithm is suitable for porting to hardware platforms to achieve algorithm acceleration.

### 4.4. FPGA Comparison Lab

In this paper, a comparison of the algorithm speed of the BGIR algorithm is carried out on a PC and hardware platform using images of various resolutions. The comparison results are shown in Table 5. The data shows that the processing speed is significantly improved after the hardware platform acceleration and optimization. For images with pixels of 1280 × 720, the processing speed reaches 33.56 ms, which is about 30 fps; for images with pixels of 640 × 480, the processing speed reaches 12.27 ms, which is about 80 fps. These results quantitatively prove the advantage of the hardware deployment of the algorithm, and the speed of the fpga-optimized version has been greatly improved compared to the software implementation.

### 4.5. ZYNQ Resource Utilization and Evaluation

This study optimizes the image-processing flow and the color separation module, significantly reducing the resource overhead of the LUT and BRAM. To achieve this, the FPGA design incorporates optimization techniques such as PIPELINE and DATAFLOW, efficiently managing the usage of logic resources while enhancing algorithm execution efficiency. Nevertheless, investigations reveal that, when processing large-size photos, the increase in computational complexity still results in a rise in BRAM utilization.

The hardware resource utilization of the proposed method is summarized in Table 6, along with key metrics such as the BRAM, DSP, and LUT. According to the data evaluation, the LUT utilization rate is 61%, primarily supporting the computationally demanding tasks in the BGIR algorithm; the DSP occupancy rate is within a reasonable range, and future expansion of the computational module may enhance the accuracy of the results; the remaining resources have lower utilization rates, and the distribution is more balanced overall.

## 5. Discussion

### 5.1. Results Discussion

By comparing and analyzing parameters such as information entropy between the proposed and existing methods, it is evident that the average values obtained by the BGIR algorithm consistently outperform those from alternative methods. Significant algorithmic speedups are achieved to meet the real-time image-processing requirements, a result that holds true when applied to both datasets and real-world data. In conclusion, the contrasted values derived from the proposed method show better performance overall compared to other methods. In terms of subjective visualization of the image, the proposed algorithm effectively improves the overall brightness and details of the image while ensuring the processing speed of the algorithm, and also effectively reduces the noise, which outperforms the other methods in terms of subjective visualization and provides an overall advantage.

### 5.2. Limitation

Although the BGIR algorithm offers several advantages over other methods, it also has some drawbacks. For example, the image process, to a certain extent, will randomly lose some detail content. The main causes include the components involved in the color space transformation process, the hardware’s use of iterative approximation leading to minor value loss, operations like exponentials in the algorithm, and the FPGA’s use of lookup table approximation, which results in the loss of weak luminance steps. Therefore, it is particularly important to study a detail extraction algorithm combined with the algorithm in this paper, such as fusion of the details extracted by the bootstrap filter, to achieve image post-compensation to increase a certain amount of resource consumption in exchange for image quality. In addition, in the process of transforming the algorithm to the hardware platform to realize the algorithm, the speed of operation was improved but lost some accuracy, caused mainly by the fixed-point quantization error. The FPGA is usually a fixed-point number, and the algorithm prototype is mostly a floating-point number, resulting in some of the image information entropy index being low. For this reason, subsequent research should focus on optimizing accuracy retention, and gradient-aware quantization (which identifies high-gradient regions based on the operator and retains higher bit widths) can be employed as a method.

## 6. Conclusions

This paper presents a Brightness-Guaranteed Image Restoration (BGIR) algorithm and its ZYNQ-based hardware implementation to address low-luminance challenges in remote sensing imagery. The proposed solution integrates an enhanced multi-scale Retinex (MSR) algorithm with gamma correction for comprehensive image quality improvement, implemented on an FPGA platform for accelerated processing.

Experimental validation demonstrates the algorithm’s effectiveness through quantitative metrics: on the software platform, BGIR achieves a Peak Signal-to-Noise Ratio (PSNR) of 22.05 and Structural Similarity Index (SSIM) of 0.88, indicating significant brightness enhancement while maintaining critical image details. The hardware-accelerated implementation on the ZYNQ platform shows substantial performance gains, processing images with 1280 × 720 resolution in 33.56 ms (≈30 fps) and 640 × 480 images in 12.7 ms (≈80 fps). These processing rates satisfy the real-time requirements for laboratory-based spatial applications.

## Figures and Tables

**Table 1:** Comparative analysis of various algorithms (640 × 480).

	HE	SSR	MSR	MSRCR	BGIR
E	7.6	7.1	7.0	7.3	6.9
PSNR	16.2	14.7	15.0	19.8	25.4
SSIM	0.58	0.52	0.55	0.75	0.88
IPCI	0.150	0.147	0.155	0.182	0.211
NIQE	5.39	4.85	3.42	2.97	3.15
EPI	0.18	0.22	0.27	0.30	0.33

**Table 2:** Comparative analysis of various algorithms (1280 × 720).

	HE	SSR	MSR	MSRCR	BGIR
E	6.5	7.0	6.9	7.2	6.3
PSNR	15.8	14.2	14.6	19.3	24.6
SSIM	0.60	0.54	0.57	0.77	0.89
IPCI	0.152	0.149	0.158	0.185	0.215
NIQE	5.12	4.68	3.55	3.06	3.23
EPI	0.17	0.20	0.27	0.29	0.32

**Table 3:** Comparative analysis of various algorithms.

	HE (ms)	SSR (ms)	MSR (ms)	MSRCR (ms)	BGIR (ms)
1280 × 720	198.6	350	1260	1420	480
640 × 480	52.1	105	520	560	225

**Table 4:** Processing outcomes for various photos.

	E	PSNR	SSIM
Reference [28]	6.38	16.74	0.48
Reference [29]	7.14	16.58	0.53
Reference [30]	7.03	16.05	0.86
Reference [9]	7.53	15.14	0.78
Reference [31]	7.32	16.61	0.84
Ours	6.85	22.05	0.88

**Table 5:** Processing results of different images.

	PC/ms	ZYNQ/ms
640 × 480	622	12.27
1280 × 720	1083	33.56

**Table 6:** ZYNQ resource usage.

	Usage	Available	Proportion
BARM	63	280	22%
LUT	32,724	53,200	61%
LUTRAM	1832	17,400	10%
FF	16,938	106,400	15%
DSP	63	220	28%

## Data Availability

Data are contained within the article.

## Abbreviations

The following abbreviations are used in this manuscript:

ZYNQ	Zynq-7000 All Programmable Soc
AI	Artificial Intelligence
BGIR	Bilateral Gamma Image Restoration
MSR	Multi-Scale Retinex
PSNR	Peak Signal-to-Noise Ratio
SSIM	Structural Similarity Index
Fps	Frames Per Second
SSR	Single-Scale Retinex
MSRCR	Multi-Scale Retinex with Color Recovery
HE	Histogram Equalization
